# Powered single hip joint exoskeletons for gait rehabilitation: a systematic review and Meta-analysis

**DOI:** 10.1186/s12891-024-07189-4

**Published:** 2024-01-20

**Authors:** Mahla Daliri, Mohammad Ghorbani, Alireza Akbarzadeh, Hossein Negahban, Mohammad H Ebrahimzadeh, Elham Rahmanipour, Ali Moradi

**Affiliations:** 1 Orthopedics Research Center, Mashhad University of Medical Sciences, Mashhad, Iran; 2 Health Policy Research Center, Shiraz University of Medical Sciences, Shiraz, Iran; 3 Mechanical Engineering Department, Ferdowsi University of Mashhad, Mashhad, Iran; 4 Department of Physical Therapy, School of Paramedical and Rehabilitation Sciences, Mashhad University of Medical Sciences, Mashhad, Iran

**Keywords:** Hip, Powered, Exoskeleton, Gait, Rehabilitation, Clinical outcomes

## Abstract

**Background:**

Gait disorders and as a consequence, robotic rehabilitation techniques are becoming increasingly prevalent as the population ages. In the area of rehabilitation robotics, using lightweight single hip joint exoskeletons are of significance. Considering no prior systematic review article on clinical outcomes, we aim to systematically review powered hip exoskeletons in terms of gait parameters and metabolic expenditure effects.

**Methods:**

Three databases of PubMed, Scopus, and Web of science were searched for clinical articles comparing outcomes of gait rehabilitation using hip motorized exoskeleton with conventional methods, on patients with gait disorder or healthy individuals. Of total number of 37 reviewed articles, 14 trials were quantitatively analyzed. Analyses performed in terms of gait spatiotemporal parameters like speed (self-speed and maximum speed), step length, stride length, cadence, and oxygen consumption.

**Results:**

Improved clinical outcomes of gait spatiotemporal parameters with hip joint exoskeletons are what our review’s findings show. In terms of gait values, meta-analysis indicates that rehabilitation with single hip joint exoskeleton enhanced parameters of maximum speed by 0.13 m/s (0.10–0.17) and step length by 0.06 m (0.05–0.07). For the remaining investigated gait parameters, no statistically significant difference was observed. Regarding metabolic parameters, oxygen consumption was lower in individuals treated with hip exoskeleton (− 1.23 ml/min/kg; range − 2.13 to − 0.32).

**Conclusion:**

Although the analysis demonstrated improvement with just specific gait measures utilizing powered hip exoskeletons, the lack of improvement in all parameters is likely caused by the high patient condition heterogeneity among the evaluated articles. We also noted in patients who rehabilitated with the hip exoskeleton, the oxygen cost was lower. More randomized controlled trials are needed to verify both the short- and long-term clinical outcomes, including patient-reported measures.

**Level of evidence:**

Level I (systematic review and meta-analysis).

**Supplementary Information:**

The online version contains supplementary material available at 10.1186/s12891-024-07189-4.

## Introduction

Gait disorders become more common as population ages, increasing from 30% of adults 60 years and older to more than 60% of people over the age of 80 [1, 2]. Gait disorders may originate from neurologic problems (e.g. sensory or motor impairments like stroke or spinal cord injuries), orthopedic disorders (e.g. osteoarthritis or skeletal deformities) or other general medical conditions (e.g. cardiovascular diseases, pulmonary diseases, obesity or psychiatric disorders) [3–5]. Gait and balance problems contribute to poor quality of life and increased morbidity and mortality in the elderly [2]. Although there are medical and surgical managements for gait disorders, ambulatory devices are the only option left in most cases [6], and previous studies supported that continues locomotor activity improves patients’ condition [7, 8].

Exoskeletons are external devices worn with the aim of rehabilitation or replacement for lost physical functions and integrate the human intelligence with robotic power [9]. In the past decade, several lower limb exoskeletons (LLEs) have been developed such as ReWalk [10], Honda Walking Assist® (HWA) [11], Stride Management Assist (SMA), Gait Enhancing Mechatronic System (GEMS), Hybrid Assistive Limb (HAL) [12] and Vanderbilt [13]. However, there is still a lot to do in development and application of LEEs, such as control, actuators, and humane-machine interface (HMI) optimization. By providing almost consistent torque profiles throughout the rehabilitation process, which cannot be accomplished by manual assistance or verbal feedback from the physiotherapist, powered exoskeletons help increase treatment reproducibility. Additionally, by integrating individualized parameters, the device power can be adjusted to target particular gait impairments in a regulated manner. Using these devices relieves therapists of huge burden while providing various personalized task-specific practice in novel dynamic environments, and they allow continuous monitoring of patients’ performance and progression [14]. The employment of light single-joint exoskeletons for gait training has recently become a trend in the field of rehabilitation robotics [15]. Motorized hip exoskeletons appear to favorably enhance the rehabilitation of stroke survivors and other patients with restricted movement capacities by enhancing gait spatiotemporal parameters, metabolic economy, and biomechanical quality [16, 17]. Additionally, patients with neurological conditions brought on by disorders or trauma like a stroke or spinal cord injury frequently have weak muscles, which could result in inadequate force or torque at the hip joints during limb movements [18], specially that due to the different muscle properties, the hip joint has a higher metabolic expenditure for the generation of equivalent mechanical joint power [19]. Fully functioning human walking lowers the risk of strokes and coronary heart disease, improving both psychological and physical well-being [20].

Today, the exoskeletons’ technology progress have been studied and reviewed comprehensively [21–23]. Regarding powered hip robots, there are some reviews in terms of design and control [22, 24]. However, we found no comprehensive review in terms of clinical gait outcomes with powered single hip joint exoskeletons (PSHJE). For a better comprehension of this technology practical applications, we extensively summarize the 1) clinical outcomes including gait spatiotemporal parameters and 2) metabolic cost on walking-assist hip exoskeletons in this systematic review.

## Materials and methods

### Protocol

This systematic review and meta-analysis was conducted based on the Preferred Reporting Items for Systematic Reviews and Meta-Analysis: the PRISMA Statement [25].

### Searching strategy

An electronic search was conducted in the databases PubMed/MEDLINE, Scopus and ISI Web of Science from inception until August 31th, 2022. The search statement was: ((“robot*” OR “exoskelet*” OR “automatic orthos*” OR “powered orthos*”) AND (“hip” OR “coxa*” OR “acetabul*”) AND (“gait” OR “walk” OR “rehab*” OR “mobil*”). The references of retrieved articles were also checked to find possibly relevant studies. We refined our search by available English language abstract and article document type. Other filters were not applied. Grey literature was not checked in this study.

### Eligibility criteria and study selection

**Fig. 1:**
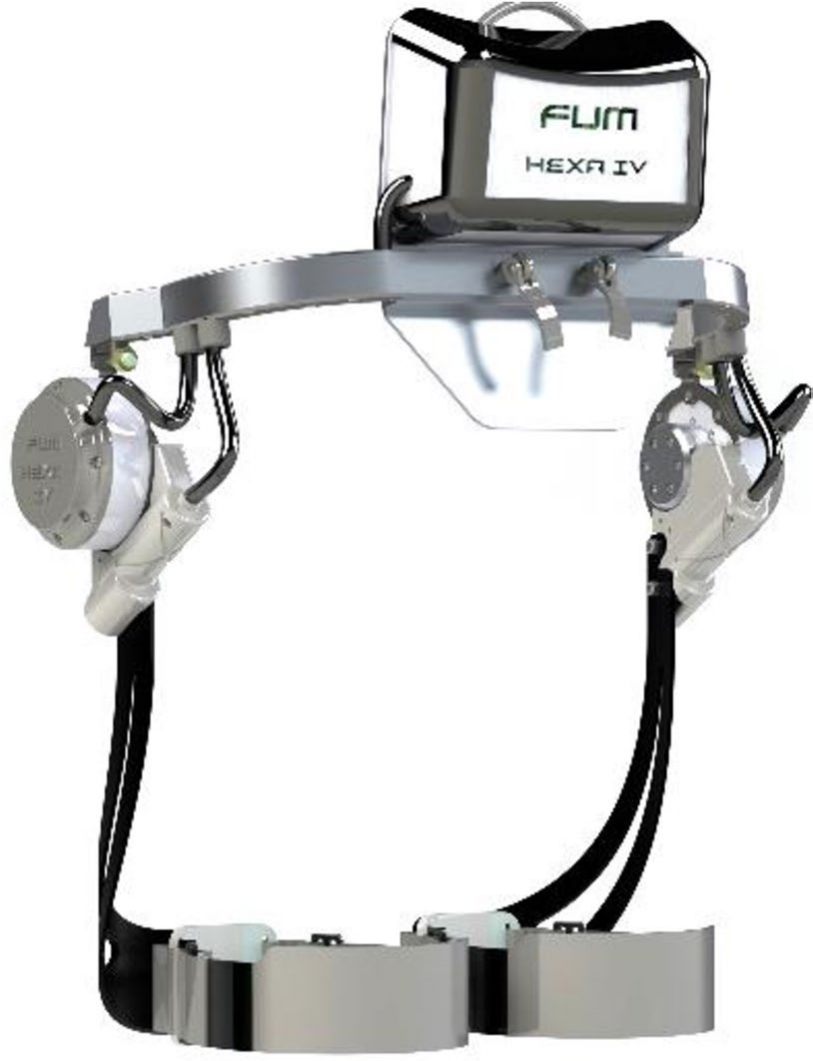
The powered single hip joint exoskeleton (PSHJE) robots developed in FUM CARE, named HEXA. The robot is portable and weighs less than 4 kg

The present systematic review screened all studies that met the PICOD protocol requirements: P (Problem): gait disorder; I (Intervention): rehabilitation with PSHJE; C (Comparison): conventional rehabilitation; O (Outcomes): gait spatiotemporal parameters; and D (design): controlled or pre-post clinical trial. All the search results were imported to Endnote X9 citation manager and duplicate studies were removed. Two reviewers (M. D, M. Gh) independently screened the titles and abstracts of studies to select relevant ones. Disagreements were resolved by consensus. Studies evaluating the outcomes of using powered single hip joint exoskeleton (PSHJE) robots (Fig. 1) in patients with gait disorders (stroke, amputees, joint arthroplasty, elderly, cerebral palsy, etc.) were included in this review. All abstracts, review articles, case-reports, case-series, letters to editors, prototypes and animal studies were excluded. Additional exclusion criteria were studies dealing with powered exoskeletons on joints other than hip or whole lower body robots, passive hip exoskeletons, and non-clinical studies (Supplementary Figure). Whenever several studies were published from a trial with the same population, only the results and data of the last study (provided that it has the largest sample size, mentions accurate and complete information of patients in depth, and a complete/correct reporting of the outcomes) would meet the criteria for our systematic review.

### Data extraction and quality assessment

Three reviewers separately collected data from full texts of included studies using a pre-designed Excel form. Results were compared and double checked by the same reviewers. The data extracted included: author, year, study design, sample size, disorder, mean age, sex ratio, disease type, robot name, self-speed (self-selected comfortable speed), max speed, cadence, step length, stride length and metabolic cost. The methodological quality and validity of each included study was evaluated using the JBI critical appraisal checklists for clinical trials and single group studies (Tables 1 and 2).

**Table 1:** Quality assessment checklist for randomized controlled trials (Critical Appraisal tools for use in JBI Systematic Reviews)

N	Criteria	Jayaraman (2018)	Ji (2018)	Kawasaki (2014)	Martini (2019)	Tanaka (2017)	Buesing (2015)	Hwang-Jae Lee (2019)
1	Was true randomization used for assignment of participants to treatment groups?	Yes	Yes	Yes	Yes	Yes	Yes	Yes
2	Was allocation to treatment groups concealed?	Unclear	Unclear	No	Unclear	No	No	Unclear
3	Were treatment groups similar at the baseline?	Yes	Yes	NA	No	Yes	Yes	Yes
4	Were participants blind to treatment assignment?	No	No	No	No	No	No	No
5	Were those delivering treatment blind to treatment assignment?	No	Unclear	No	Unclear	No	No	Unclear
6	Were outcomes assessors blind to treatment assignment?	Yes	Unclear	No	Unclear	No	Yes	Unclear
7	Were treatment groups treated identically other than the intervention of interest?	Yes	Yes	Yes	Yes	Yes	Yes	Yes
8	Was follow up complete and if not, were differences between groups in terms of their follow up adequately described and analyzed?	Yes	Yes	Yes	Yes	Yes	Yes	NA
9	Were participants analyzed in the groups to which they were randomized?	Yes	Yes	Yes	Yes	Yes	Yes	Yes
10	Were outcomes measured in the same way for treatment groups?	Yes	Yes	Yes	Yes	Yes	Yes	Yes
11	Were outcomes measured in a reliable way?	Yes	Yes	Yes	Yes	Yes	Yes	Yes
12	Was appropriate statistical analysis used?	Yes	Yes	Yes	Yes	Yes	Yes	Yes
13	Was the trial design appropriate, and any deviations from the standard RCT design (individual randomization, parallel groups) accounted for in the conduct and analysis of the trial?	Yes	Unclear	No	Yes	Yes	Yes	Yes

*NA* Not applicable

**Table 2:** Quality assessment checklist for single group and non-randomized clinical trials (Critical Appraisal tools for use in JBI Systematic Reviews)

N	Criteria	Kitatani (2014)	Koseki (2021)	Kim (2018)	Hwang-Jae Lee (2016)	Su-Hyun Lee (2017)	Lenzi (2013)
1	Were there clear criteria for inclusion in the case series?	Yes	Yes	Yes	Yes	Yes	No
2	Was the condition measured in a standard, reliable way for all participants included in the case series?	NA	Yes	Yes	Yes	Yes	NA
3	Were valid methods used for identification of the condition for all participants included in the case series?	NA	Yes	Yes	Yes	Yes	NA
4	Did the case series have consecutive inclusion of participants?	Yes	Yes	Yes	Yes	Yes	No
5	Did the case series have complete inclusion of participants?	Yes	Yes	Yes	Yes	Yes	No
6	Was there clear reporting of the demographics of the participants in the study?	Yes	Yes	Yes	Yes	Yes	No
7	Was there clear reporting of clinical information of the participants?	NA	Yes	Yes	Yes	Yes	No
8	Were the outcomes or follow up results of cases clearly reported?	Yes	Yes	Yes	Yes	Yes	Yes
9	Was there clear reporting of the presenting site(s)/clinic(s) demographic information?	Yes	Yes	Yes	Yes	Yes	Yes
10	Was statistical analysis appropriate?	Yes	Yes	Yes	Yes	Yes	Yes

*NA* Not applicable

### Statistical analysis

Our primary focus are clinical outcomes in terms of gait spatiotemporal parameters. As the secondary outcomes we reviewed metabolic and oxygen cost, hip joint angle, and muscle activity. Forest plots were depicted to assess for heterogeneity and calculate the pooled weighted mean difference (WMD) with corresponding 95% confidence intervals for visual inspection across studies. Due to conceptual heterogeneity, a random-effects meta-analysis was conducted to account for the heterogeneity of the study populations. Pooled estimates with their corresponding 95% CIs were calculated using inverse-variance weights methods [26]. The I^2^ statistics was used to assess the heterogeneity across studies (I2 = 0% indicates no observed heterogeneity and I2 ≥ 50% indicates substantial heterogeneity). Cochran’s Q statistic was also used to analyze the statistical significance of heterogeneity [27]. Sensitivity analysis was performed to determine which study (if any) had the largest impact on the heterogeneity and to assess the robustness of pooled estimates. WMD was plotted against the inverse of the square of the standard error. All statistical tests were two-tailed, and the significance level was set at less than 0.05 for all, except for the heterogeneity test. Statistical analyses were performed using Stata version 17.0 (Stata Corp., College Station, Texas, USA).

## Results

### Study characteristics

Finally, a systematic review and meta-analysis of 13 comparative clinical research, including 7 clinical trials and 7 single group trials was conducted. Four publications assessed GEMS, four SMA, two HWA, and two Active Pelvis Orthosis (APO). Each of the following was examined in a single paper: ALEX II exoskeleton, HAL lumbar type, and Robotic Assisted Rehabilitation Trainer (RART). One study focused on pediatric patients [28], while others investigated adults and the elderly (Table 3).

**Table 3:** Included studies’ basic characteristics

N	Author (year)	Study type	Sample size	Condition	F/U (month)	Group (N)	Robot	Age	Sex ratio (female %)
1	Jayaraman (2018)	RCT	50	Chronic stroke	3	PSHJE (25)	SMA	59.5 ± 9.7	36
						No PSHJE (25)		61.6 ± 12.6	32
2	Kitatani (2014)	Single group clinical trial	10	Healthy	0	PSHJE	SMA	24.4 ± 3.5	50
						No PSHJE			
3	Koseki (2021)	NRCT	22	Knee arthroplasty	2	PSHJE (11)	HWA	71.8 ± 6.2	100
						No PSHJE (11)		75.9 ± 6.9	90.9
4	Ji (2018)	RCT	16	Hemiplegic	2	PSHJE (8)	RART	48. 85 ± 20.9	43.75
						No PSHJE (8)			
5	Kim (2018)	Single group clinical trial	15	Healthy elderly	0	PSHJE	GEMS	74.33 ± 4.56	60
						No PSHJE			
6	Kawasaki (2020)	Cross-over RCT	10	Spastic CP children	0	PSHJE	HWA (RAGT)	11.1 ± 2.3	60
						No PSHJE			
7	Hwang-Jae Lee (2016)	Single group clinical trial	30	Healthy elderly	0	PSHJE	GEMS	74.07 ± 4.14	50
						No PSHJE			
8	Su-Hyun Lee (2017)	Single group clinical trial	30	Healthy elderly	0	PSHJE	GEMS	74.10 ± 4.18	53.3
						No PSHJE			
9	Hwang-Jae Lee (2019)	RCT	26	Chronic stroke	0	PSHJE (14)	GEMS	61.85 ± 7.87	50
						No PSHJE (12)		62.25 ± 6.36	41.6
10	Lenzi (2013)	Single group clinical trial	10	Healthy	0	PSHJE	ALEX II	NI	NI
						No PSHJE			
11	Martini (2019)	RCT	20	Elderly	1	PSHJE (10)	APO	70 ± 5	20
						No PSHJE (10)		70 ± 4	70
12	Tanaka (2017)	RCT	41	subacute stroke patients with hemiplegia	0	PSHJE (21)	SMA	64.9 ± 12.2	38
						No PSHJE (20)		62.3 ± 9.3	30
13	Buesing (2015)	RCT	50	Chronic stroke	3	PSHJE (25)	SMA	60 ± 2	32
						No PSHJE (25)		62 ± 3	36

*F/U* follow up, *RCT* randomized clinical trial, *NRCT* non-randomized clinical trial, *PSHJE* powered single hip joints exoskeleton, *SMA* Stride Management Assist, *HWA* Honda Walking Assist, *RART* Robotic Assisted Rehabilitation Trainer, *GEMS* Gait Enhancing and Motivating System, *RAGT* Robot-assisted gait training, *APO* Active Pelvis Orthosis, *HAL* Hybrid Assistive Limb, *NI* Not indicated

### Qualitative results

#### Clinical outcomes including gait spatiotemporal parameters (Table 4)

When rehabilitating with Honda Walking Assist (HWA), patients with walking difficulty showed augmented motivation as measured by Intrinsic Motivation Inventory (IMI) [29]. Implementing on 50 chronic stroke patients, powered hip exoskeleton could enhance gait clinical outcomes (endurance (P .033), balance (P 0.036), step count (P 0.013) etc.), compared with the functional training [17]. Patient-reported outcomes of balance confidence and falls efficacy also improved with hip exoskeleton [17]. Assistance timing increases, step length, cadence, and walk ratio (i.e. step length/cadence ratio) all increase with Gait Enhancing Mechatronic System (GEMS) [30]. Healthy elderly patients showed no difference in stair climbing cadence while using GEMS or not [31]. Studying hip exoskeleton in comparison with treadmill on children with cerebral palsy (CP), showed notable increase in walking speed (*P* < 0.05) [28]. In one patient with spinal cord injury, powered hip exoskeleton increased walking speed (0.24 to 0.31 m/s), step length (0.38 to 0.41 m), cadence (37.5 to 44.81 step/min), and decreased compensatory movements, when compared with Isocentric Reciprocating Gait Orthosis (IRGO) [32]. With relation to integrating orthotics along with functional electrical stimulation (FES), reciprocal gait orthosis (RGO) with a variable constraint hip mechanism (VCHM) compared with IRGO on five able-bodied individuals, and stated that when hip controller was active, hip kinematics was more similar to normal hip joints (ICC = 0.96). There was no difference in terms of step length, but IRGO resulted in walking speed closer to the norms [33]. Compared to the IRGO (intraclass correlation coefficient = .68), the VCHM with controller active enabled the production of joint moments that were closer to the normal values (ICC = 0.80) [33]. During a randomized trial, the robotic trainer to control hip motions, improved gait parameters of speed, cadence, and balance in hemiplegic patients [34]. Koseki et al. conducted three studies on Honda Walking Assistive device® (HWA) and showed increased gait parameters (speed, step length and cadence) in one subject with hip osteoarthritis and two subjects with transfemoral amputation [11, 35]. They also noted early gait improvement when waring HWA during a clinical trial and a case report on total knee arthroplasty patients [36, 37]. HWA outcomes were similar on one patient with spinal cord injury [38]. Elderly people in the GEMS group showed enhanced gait performance, reduced muscle effort, and lower metabolic expenditure [39, 40], as well as patients with chronic stroke patients [41]. Miura et al. reported enhanced balancing function variables, despite the fact that mobility function metrics like the 10MWT did not significantly improve after Hybrid Assistive Limb (HAL) physiotherapy in patients with locomotive syndrome [42]. Unlike control group, applying stride management assist (SMA) with exoskeleton resulted in notable improvement post-training in terms of maximum gait speed, paralysis-side step length, symmetry, and cadence in subacute stroke [43]. At various times, SMA group showed further advancements across gait metrics [16].

**Table 4:** Included studies’ outcomes regarding gait parameters

N	Author (year)	Group (N)	Speed (m/s) SS	Speed (m/s) MaxS	Step length (m)	Stride length (cm)	Cadence (step/min)	Fugl-Meyer (balance)	Metabolic (W/kg)	Oxygen (ml/min/kg)
1	Jayaraman (2018)	PSHJE (25)	0.95 ± 0.06	1.24 ± 0.05				21.4 ± 16.7		
		No PSHJE (25)	0.85 ± 0.04	1.12 ± 0.05				11.8 ± 16.6		
2	Kitatani (2014)	PSHJE	1.06 ± 0.16	1.51 ± 0.15						0.13 ± 0.01
		No PSHJE	1.07 ± 0.15	1.52 ± 0.13						0.15 ± 0.015
3	Koseki (2021)	PSHJE (11)	0.96 ± 0.17	1.20 ± 0.21	0.55 ± 0.08		104.1 ± 8.47			
		No PSHJE (11)	0.7 ± 0.29	0.90 ± 0.35	0.46 ± 0.11		87.53 ± 23.99			
4	Ji et al. (2018)	PSHJE (8)	0.23 ± 0.11				52.75 ± 14.65	24 (18–32)		
		No PSHJE (8)	0.29 ± 0.10				55.63 ± 5.97	18 (16–22)		
5	Kim et al. (2018)	PSHJE					53.25 ± 4.56		6.10 ± 1.15	17.43 ± 4.09
		No PSHJE					53.92 ± 5.82		6.79 ± 1.18	19.07 ± 4.00
6	Kawasaki et al. (2020)	PSHJE	0.60 ± 0.38							
		No PSHJE	0.41 ± 0.19							
7	Hwang-Jae Lee et al. (2016)	PSHJE	0.040 ± 0.001			117.76 ± 12.95	111.32 ± 9.43		4.48 (3.58–5.6)	13.5 (10.5–16)
		No PSHJE	0.035 ± 0.005			107.23 ± 15.56	105.61 ± 5.46		5.04 (3.92–6-16)	14.5 (11.5–17)
8	Su-Hyun Lee et al. (2017)	PSHJE	1.10 ± 0.13			117.76 ± 12.95	113.36 ± 6.92			
		No PSHJE	0.97 ± 0.15			107.23 ± 15.56	107.90 ± 5.80			
9	Hwang-Jae Lee et al. (2019)	PSHJE (14)	0.91 ± 0.15			92.64 ± 15.46	106.88 ± 29.48			16 ± 1.2
		No PSHJE (12)	0.78 ± 0.11			80.64 ± 16.44	88.01 ± 22.82			17.5 ± 1.5
10	Lenzi et al. (2013)	PSHJE					101.4 ± 0.46			
		No PSHJE					102.7 ± 0.48			
11	Martini et al. (2019)	PSHJE (10)							3.45 ± 0.64	10.06 ± 1.88
		No PSHJE (10)							3.58 ± 0.72	10.54 ± 2.11
12	Tanaka et al. (2017)	PSHJE (21)		1.08 ± 0.43	0.53 ± 0.14		116.56 ± 24.66			
		No PSHJE (20)		1.06 ± 0.61	0.52 ± 0.19		108.80 ± 32.78			
13	Buesing et al. (2015)	PSHJE (25)	0.87 ± 0.05	1.23 ± 0.07	0.58 ± 0.03	110 ± 6	95.67 ± 3.69			
		No PSHJE (25)	0.88 ± 0.07	1.08 ± 0.07	0.52 ± 0.02	97 ± 5	97.37 ± 5.08			

*SS* self-speed, *MaxS* maximum speed, *PSHJE* powered single hip joints exoskeleton

#### Muscle activity

When adding hip joint paretic side corrective force to the robotic treadmill on 15 post stroke subjects, increased muscle activity and more symmetric hip movements were observed [44]. Corticomotor excitability (CME) corresponding to rectus femoris muscle in patients with chronic stroke augmented with hip exoskeleton compared with functional training (P 0.010). primary sensorimotor cortex (SMC) showed augmented activation in patients with stroke, as revealed by infrared spectroscopy [45].

#### Hip joint angles

Comparing powered hip exoskeleton with Isocentric Reciprocating Gait Orthosis (IRGO), hip angles were comparable to those shown by normal walking motions while wearing this orthosis, but reduced compared to normal gait with both orthoses [32]. The VCHM with controller active enabled greater hip flexion compared to the IRGO and provided smooth control of the hip joints via context-dependent coupling [33]. A case report study revealed that pneumatic artificial muscle (PAM) powered hip orthosis, operated via a voluntary activation algorithm relies on the hip joint’s angular characteristics, could be adjusted to provide a satisfying and comfortable application during the gait cycle and improved left step transposition on the patient with polio [46]. Peak hip and knee flexion angles improved (reduced) with robotic trainer to control hip motions [34]. Limb symmetry and maximum hip angle associate with hip exoskeleton assistance and timing in children with spastic CP [28]. Maximum hip flexion decreased from 45.7 to 34.4 with HWA in a patient underwent total hip arthroplasty (THA) [35] but increased from 24 to 30 in two amputees [11]. There is the report stating powered or unpowered conditions when using hip exoskeleton are similar in terms of hip moment pattern and despite having different hip joint angles for a particular walking pace, people follow similar joint moment patterns when walking [47].

#### Metabolic

Physiologic cost index (PCI) decreased 20.5% while wearing hip exoskeleton by an above knee amputee (*P* < 0.01) [48]. The association between assistant timing of GEMS hip exoskeleton and metabolic cost has shown maximum 21% reduction at 0% assistance timing compared with no exoskeleton walking [30]. GEMS was studied on healthy elderly individuals walking stairs and revealed notable decrease in oxygen consumption per unit mass (P 0.013), metabolic power per unit mass (P 0.001) and metabolic equivalents (*P* < 0.05) values [31]. When assisting hip flexion and extension of healthy individuals, oxygen consumption and heart rate reduced [49]. At a self-selected speed, GEMS resulted in a 7 and 6.6% reduction in oxygen consumption per unit and energy expenditure, respectively (p 0.05) [39], and the net cardiopulmonary metabolic energy cost was also decreased by 14.71% following the intervention in patients with chronic stroke [41]. The motorized hip exoskeleton’s interface design optimization and its effect on metabolic cost has been studied, recently [50]. Studying the Active Pelvis Orthosis (APO) on elderly, oxygen (4.24 ± 2.57%) and metabolic (− 26.6 ± 16.1%) consumption reduced post-training notably more than control group [51]. On patients with lower limb amputation, motorized hip exoskeleton could reduce 15.6% of metabolic cost [52]. When studying the effect of placing actuators on lower extremity joints, the motors at the hip were mostly responsible for the lowering of metabolic costs [53]. Hip exoskeleton from Samsung GEMSv2 decreased metabolic cost by 13.5, 15.5 and 9.8%. (31.9, 51.6 and 45.6 W) at, 0, 5, and 10% surface gradient, respectively [54]. Hip flexion and extension metabolic costs were lowered by 9.7 and 10.3%, respectively, in the optimized powered condition compared to the unpowered condition [55].

### Quantitative results (meta-analysis)

#### Gait self-speed (m/s)

**Fig. 2:**
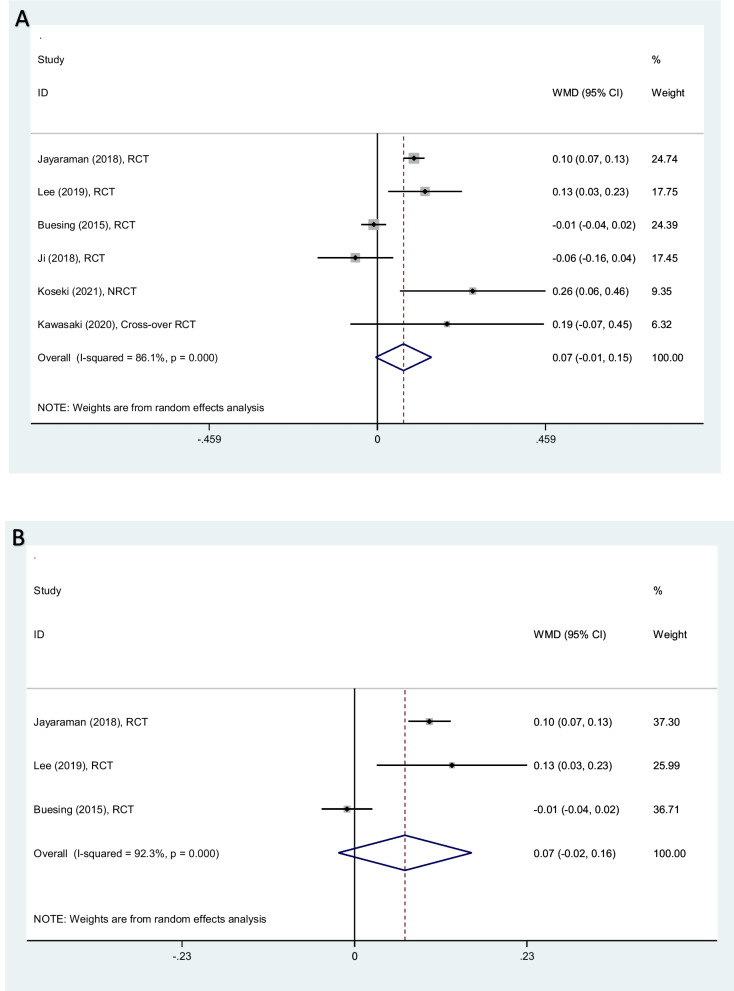
Forest plot comparing gait self-speed (m/s) between PSHJE and no PSHJE groups (**A**). Forest plot of subgroup analysis among patients with chronic stroke, comparing gait self-speed (m/s) between PSHJE and no PSHJE groups (**B**). Diamond represents the weighted mean difference (pooled WMD) estimate and its width shows corresponding 95% CI with random effects estimate. The size of the square and its central point reflects the study specific statistical weight (inverse of variance) and point estimate of the WMD and horizontal line reflects corresponding 95% CI of the study. I2 test and Cochran’s Q statistic were used to assessing the statistical heterogeneity (*P* < 0.10) across studies

Analyzing six studies, gait self -speed increased 0.07 m/s (− 0.01–0.15) on average, among patients rehabilitated by PSHJE (Fig. 2A). Sensitivity analysis showed the mean change of gait self -speed was consistent (range of summary WMDs: 0.05–0.09), indicating that the meta-analysis model was robust. I square showed high heterogeneity among reported data for gait self –speed (I^2^: 86%, *P* < 0.001). *Subgroup analysis:* among patients with chronic stroke, three clinical trials were analyzed. The results of subgroup meta-analyses show that gait self -speed increased 0.07 m/s (− 0.02–0.16) on average. I square also showed high heterogeneity in subgroup meta-analysis (I^2^: 92%, *P* < 0.001) (Fig. 2B).

#### Gait max speed (m/s)

**Fig. 3:**
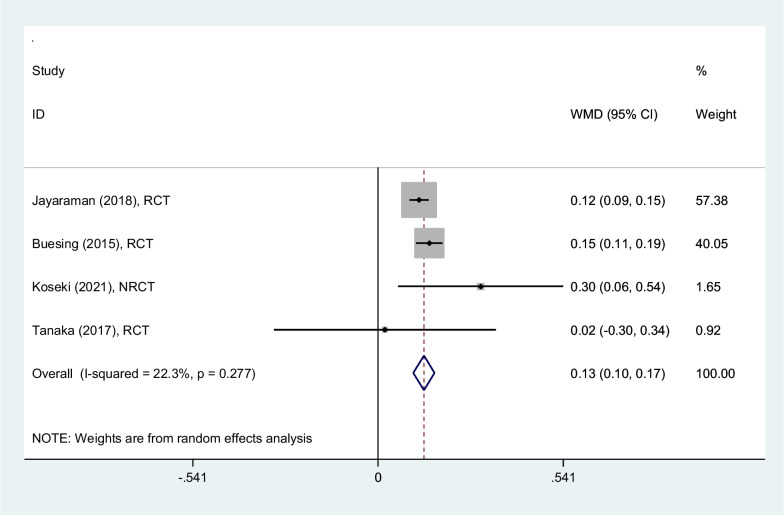
Forest plot comparing gait maximum-speed (m/s) between PSHJE and no PSHJE. Diamond represents the weighted mean difference (pooled WMD) estimate and its width shows corresponding 95% CI with random effects estimate. The size of the square and its central point reflects the study specific statistical weight (inverse of variance) and point estimate of the WMD and horizontal line reflects corresponding 95% CI of the study. I2 test and Cochran’s Q statistic were used to assessing the statistical heterogeneity (*P* < 0.10) across studies

Analyzing four studies, gait max speed increased 0.13 m/s (0.10–0.17) on average, among patients rehabilitated by PSHJE. Sensitivity analysis showed the mean change of gait max speed was consistent (range of summary WMDs: 0.13–0.15), indicating that the meta-analysis model was robust. I square showed low heterogeneity among reported data for gait max speed (I^2^: 22%, *P* = 0.28) (Fig. 3).

#### Step length (m)

**Fig. 4:**
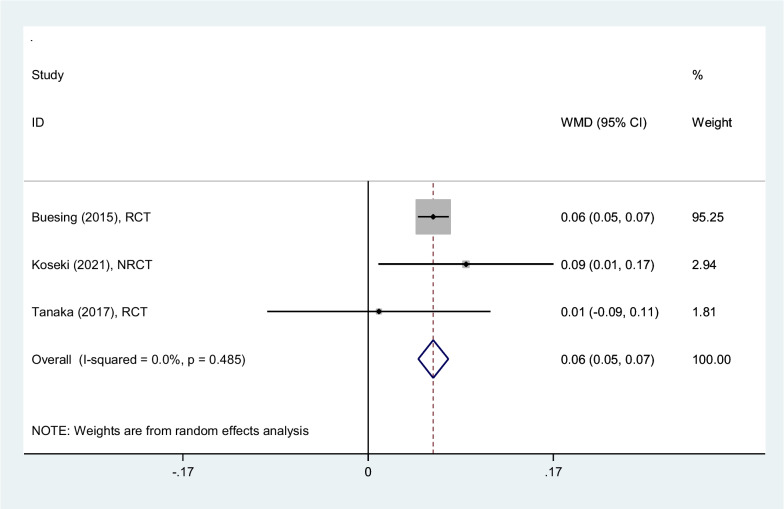
Forest plot comparing step length (m) between PSHJE and no PSHJE groups. Diamond represents the weighted mean difference (pooled WMD) estimate and its width shows corresponding 95% CI with random effects estimate. The size of the square and its central point reflects the study specific statistical weight (inverse of variance) and point estimate of the WMD and horizontal line reflects corresponding 95% CI of the study. I2 test and Cochran’s Q statistic were used to assessing the statistical heterogeneity (*P* < 0.10) across studies

Analyzing three studies, step length increased 0.06 m (0.05–0.07) on average, among patients rehabilitated by PSHJE. Sensitivity analysis showed the mean change of step length was consistent (range of summary WMDs: 0.05–0.06), indicating that the meta-analysis model was robust. I square showed low heterogeneity among reported data for step length (I^2^: 0%, *P* = 0.48) (Fig. 4).

#### Cadence (step/min)

**Fig. 5:**
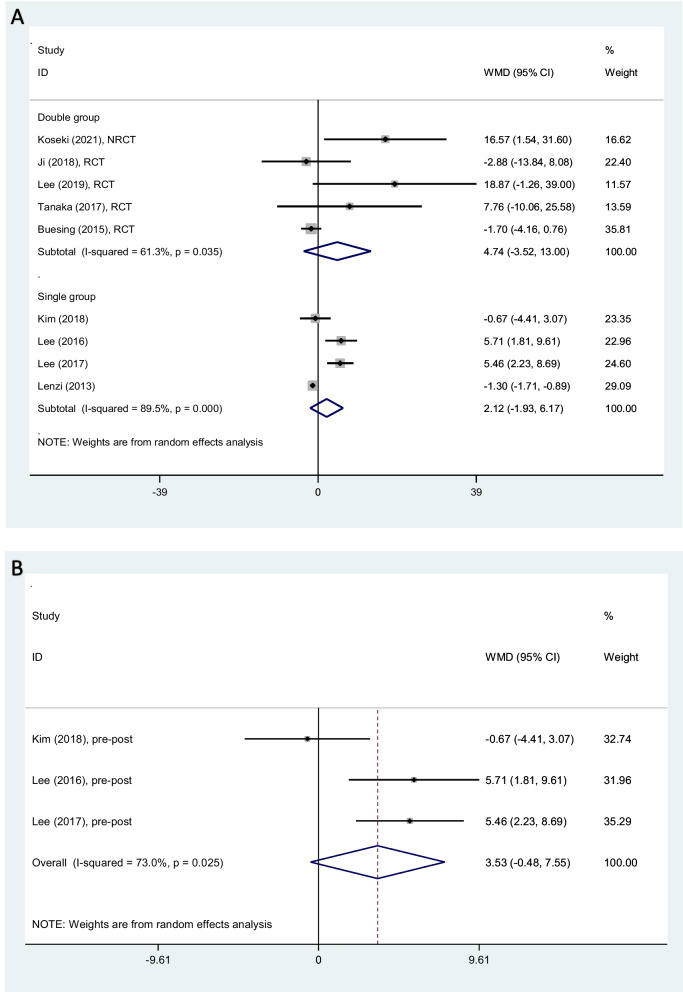
Forest plot comparing cadence (step/min) between PSHJE and no PSHJE groups (**A**). Forest plot of subgroup analysis among healthy elderly individuals, comparing cadence (step/min) between PSHJE and no PSHJE groups (**B**). Diamond represents the weighted mean difference (pooled WMD) estimate and its width shows corresponding 95% CI with random effects estimate. The size of the square and its central point reflects the study specific statistical weight (inverse of variance) and point estimate of the WMD and horizontal line reflects corresponding 95% CI of the study. I2 test and Cochran’s Q statistic were used to assessing the statistical heterogeneity (*P* < 0.10) across studies

Analyzing five clinical trials, cadence increased 4.74 step/min (− 3.52–13.00) on average, among patients rehabilitated by PSHJE (Fig. 5A). I square showed high heterogeneity among reported data for cadence (I^2^: 61%, *P* = 0.035). Sensitivity analysis showed the mean change of cadence was not consistent (range of summary WMDs: 1.1–8.5), indicating that the meta-analysis model was not robust.

Analyzing four single group pre-post studies, cadence increased 2.12 step/min (− 1.93–6.17) on average, among patients rehabilitated by PSHJE (Fig. 5A). I square showed high heterogeneity among reported data for cadence (I^2^: 89%, *P* < 0.001). Sensitivity analysis showed the mean change of cadence was not consistent (range of summary WMDs: 0.94–3.5), indicating that the meta-analysis model was not robust.

*Subgroup analysis:* among healthy elderly, three single group pre-post studies were analyzed. The results of subgroup meta-analyses show that cadence increased 3.53 step/min (− 0.48–7.55) on average (Fig. 5B). I square showed high heterogeneity among reported data for cadence (I^2^: 73%, *P* = 0.025). Sensitivity analysis showed the mean change of cadence was not consistent (range of summary WMDs: 2.5–5.5), indicating that the meta-analysis model was not robust.

#### Oxygen (ml/min/kg)

**Fig. 6:**
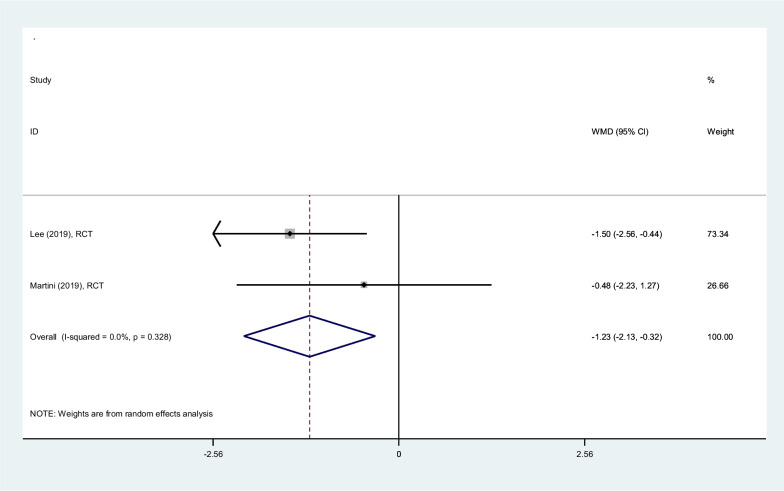
Forest plot comparing oxygen cost (ml/min/kg) between PSHJE and no PSHJE groups. Diamond represents the weighted mean difference (pooled WMD) estimate and its width shows corresponding 95% CI with random effects estimate. The size of the square and its central point reflects the study specific statistical weight (inverse of variance) and point estimate of the WMD and horizontal line reflects corresponding 95% CI of the study. I2 test and Cochran’s Q statistic were used to assessing the statistical heterogeneity (*P* < 0.10) across studies

Pooled estimation of the two RCTs, comparing PSHJE rehabilitation with control group, oxygen cost decreased − 1.23 ml/min/kg (− 2.13 to − 0.32) on average, among patients rehabilitated by PSHJE (Fig. 6). I square showed low heterogeneity among reported data for oxygen cost (I^2^: 0%, *P* = 0.33).

## Discussion

### Background

The present review investigated the association of the motorized hip-assist robots’ application with gait and metabolic parameters in patients with gait disorders, as well as healthy individuals. The main meta-analysis finding was that two of the five gait spatiotemporal values for max speed and stride length improve significantly after rehabilitation with single joint hip powered robots compared with conventional methods. Additionally, oxygen consumption was lower in individuals used hip robots. Gait values of self-peed and step length were also different between two groups but not statistically significantly (*P* = 0.09 and 0.12, respectively).

### Limitation

The included studies typically have a small sample size, and limited follow-up period. Therefore, until more consistent results are revealed in bigger RCTs, no clinical advice can be made to use hip exoskeletons in people with gait disability. The patient’s condition heterogeneity among analyzed articles are significant. However, this does not seem to be the source of heterogeneity (high I^2^) in the gait self-speed and cadence, because the subgroup analyses on the homogenous population (chronic stroke for gait self-speed and healthy elderly individuals for cadence) did not affect the I^2^ value.

### Discussion

Gait speed (*n* = 12) and cadence (*n* = 10) were the most often reported gait parameters, even though almost all included studies examined at least one temporal gait measure. Gait speed is significant for out-door everyday life activities, as well as enrolling in a rehabilitation program. Unfortunately, there is a lack of agreement regarding the best biomechanical gait measurements to utilize when examining motor coordination and the quality of walking patterns [56–58]. Although quantitative analysis in this study showed some gait values were not significantly different between the two groups, hip exoskeleton provides the opportunity for the patient to walk on his own and correct gait motor errors, and therefore increase their self-confidence, motivation, and improve psychological status.

The neuroplasticity phenomenon is the primary mechanism in which exoskeletons correct gait pattern. A well-designed robot should be able to produce complex, controlled multimodal stimulation from the bottom up and top down in order to alter the plasticity of brain connections through movement [59]. In patients with gait disorders, brain neurophysiology and organization are altered, leading to distinct brain activity patterns from those of healthy people [60]. Humans have a central pattern generator (CPG) that uses supra-spinal control of cerebral neural networks to enable rhythmic and repeated locomotor patterns [61]. Specific abnormalities in human movement are brought on by damage to specific supra-spinal structures, as seen in people who have had brain injuries such strokes [62]. To encourage the restoration of a movement pattern, the exoskeleton should provide flexibility in lower limb dynamics through sufficient degrees of freedom in all three motion planes [63]. Multisensory stimulation has been shown to be beneficial for brain reconfiguration. The reconfiguration is provided by the combination of personalized support and progression, real-time monitoring and instruction, and motor exercises that test balance control and coordination. Furthermore, because of the greater weight-shift to the affected (weak) leg during the corrective sessions, the diseased muscles become substantially more active [64, 65].

Future research should focus on improving methods for determining each patient’s specific power needs while moving through a powered single joint hip exoskeleton and matching those needs to a practical and production of smaller powered actuators for this use. As a result, patients’ compliance toward lower weight exoskeletons would increase. According on the user’s physical conditions, the rehabilitation process for people with gait abnormalities using powered hip exoskeletons can be separated into multiple stages. After a prolonged period of use, the user’s gait function may improve if they remove their exoskeleton. Nevertheless, after its discontinuation, there is a chance that gait pattern goes back to how it was. Therefore, to conduct a more robust homogeny meta-analysis on clinical outcomes, more randomized controlled trials must be carried out on each of the gait disorder types. These trials might confirm both the short- and long-term clinical outcomes, including patient-reported satisfaction, quality of life, and fall prevention on individuals with gait abnormalities. Evaluating post-intervention satisfaction and its association with gait outcomes would be an interesting topic to explore in future studies. By assessing patient satisfaction, we can gain insights into their subjective experiences and perceptions of the robotic rehabilitation interventions. This information can provide valuable supplementary data to further understand the effectiveness and acceptability of these interventions.

### Conclusion

Analyzing current literature on powered hip exoskeleton, gait max speed and stride length were different between the two groups (*P* < 0.05). Regarding metabolic cost, patients who rehabilitated with the hip exoskeleton have consumed lower amounts of oxygen. More randomized controlled trials must be carried out to verify both the short- and long-term clinical outcomes, including patient-reported measures. The consideration of population demographic and ethnic diversity among the included articles is an important limitation to be acknowledged.

### Supplementary Information

**Additional file 1.**

## Data Availability

The datasets used and/or analyzed during the current study available from the corresponding author on reasonable request.
