# Supplementary material for: Powered single hip joint exoskeletons for gait rehabilitation: a systematic review and Meta-analysis
Source: BMC Musculoskelet Disord. 2024 Jan 20;25:80. doi: 10.1186/s12891-024-07189-4 (PMC10799403; doi:10.1186/s12891-024-07189-4)
Supplement: Supplementary file 1 — Additional file 1. [file 12891_2024_7189_MOESM1_ESM.pdf]

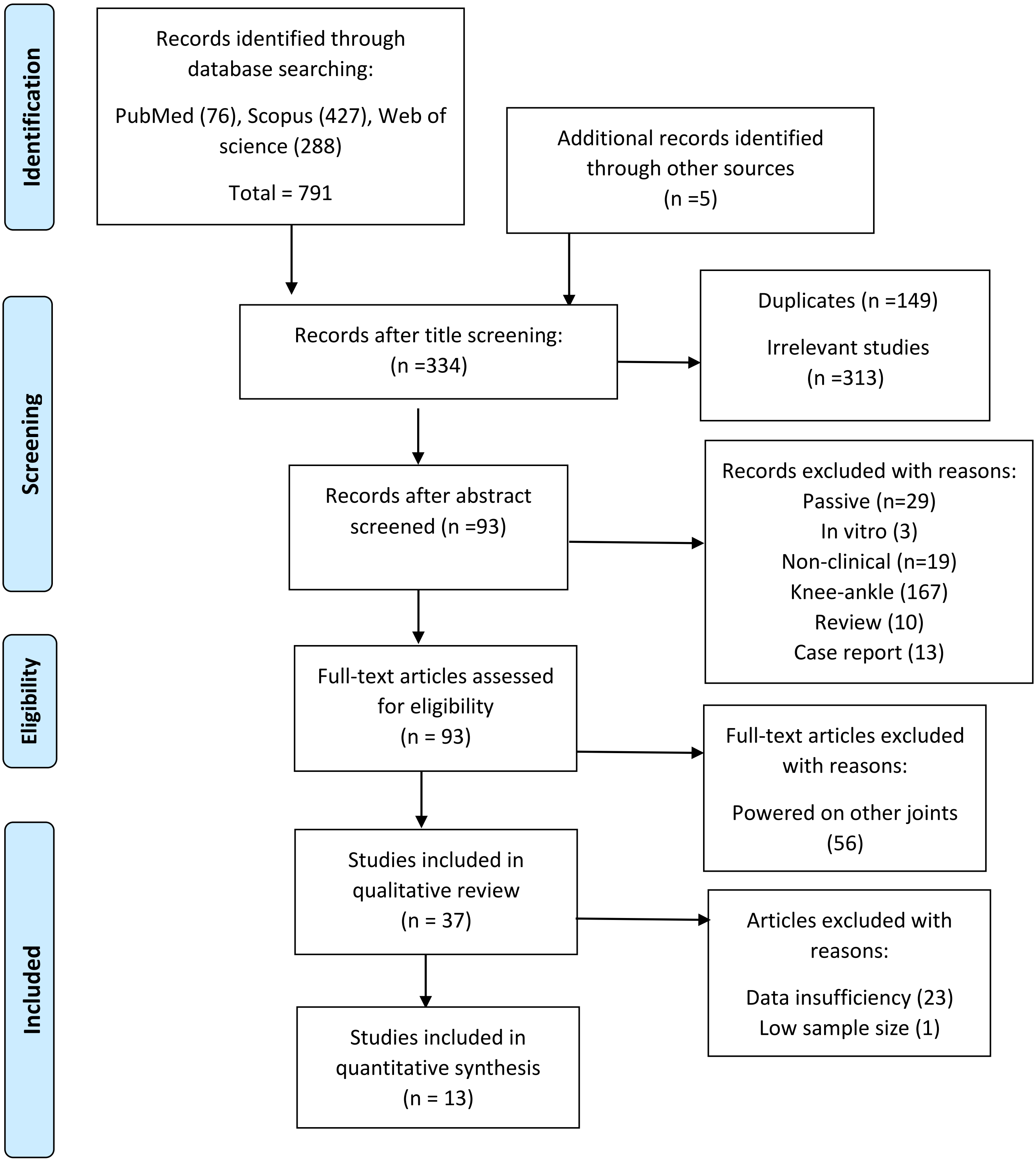

Supplementary Figure: Flowchart displaying the number of documents eliminated at each stage and the systematic search approach used.
